# Effects of imperatorin on scopolamine-induced cognitive impairment and oxidative stress in mice

**DOI:** 10.1007/s00213-014-3728-6

**Published:** 2014-09-05

**Authors:** Barbara Budzynska, Anna Boguszewska-Czubara, Marta Kruk-Slomka, Krystyna Skalicka-Wozniak, Agnieszka Michalak, Irena Musik, Grazyna Biala

**Affiliations:** 1 Department of Pharmacology and Pharmacodynamics, Medical University of Lublin, Lublin, Poland; 2 Department of Medical Chemistry, Medical University of Lublin, Lublin, Poland; 3 Department of Pharmacognosy with Medicinal Plants Unit, Medical University of Lublin, Lublin, Poland

**Keywords:** Alzheimer, Animal model, Antioxidant, Scopolamine

## Abstract

**Rationale:**

Imperatorin, a naturally occurring furanocoumarin, inactivates gamma-aminobutyric acid transaminase and inhibits acetylcholinesterase activity.

**Objectives:**

The purpose of our experiment was to examine the influence of imperatorin on cognitive impairment and oxidative stress in the brain induced by scopolamine in male Swiss mice.

**Methods:**

In the present studies, we used scopolamine-invoke memory deficit measured in passive avoidance (PA) paradigm as an animal model of Alzheimer disease (AD).

**Results:**

Our finding revealed that imperatorin administered acutely at the doses of 5 and 10 mg/kg prior to the injection of scopolamine (1 mg/kg) improved memory acquisition and consolidation impaired by scopolamine. Furthermore, repeatable (7 days, twice daily) administration of the highest dose of imperatorin (10 mg/kg) significantly attenuated the effects of scopolamine on memory acquisition, whereas the doses of 5 and 10 mg/kg of this furanocoumarin were effective when memory consolidation was measured. Imperatorin, administered with scopolamine, increased antioxidant enzymes activity and decreased concentration of malondiamide, an indicator of lipid peroxidation level.

**Conclusions:**

These results demonstrate that imperatorin may offer protection against scopolamine-induced memory impairments and possesses antioxidant properties, thus after further preclinical and clinical studies this compound may provide an interesting approach in pharmacotherapy, as well as prophylactics of AD.

## Introduction

Imperatorin [9-(3-methylbut-2-enyloxy)-7H-furo [3,2-g]chromen-7-one] is a naturally occurring furanocoumarin, which can be found in citrus fruits, umbelliferous vegetables, and selected herbal medicines, namely in the roots and fruits of *Angelica dahurica* and *Angelica archangelica* (Umbelliferae, Apiaceae) (Baek et al. 2000). In recent years, much of the interest in this compound has centered on its pharmacological activities. Many experimental studies have shown that imperatorin possess anti-inflammatory and antitumor properties (Abed et al. 2001; Ban et al. 2003; Garcia-Argaez et al. 2000; Kawaii et al. 2001), and shows anti-HIV influences (Sancho et al. 2004) and anticonvulsant action (Łuszczki et al. 2007). Moreover, our previous study revealed that acute and repeated administration of imperatorin improves different stages of memory processes (both acquisition and consolidation) in the modified elevated plus maze test (mEPM), as well as cognitive functions when coadministered with nicotine used at the non-active dose (Budzynska et al. 2012, 2013). We also observed that this coumarin, when administered acutely and repeatedly, exerts anxiolytic effects on mice measured in the EPM test (Budzynska et al. 2012). The mechanism of action of imperatorin is connected with the inhibition of voltage-dependent calcium channels and with the blockade of serotonin (5-HT) receptors (He et al. 2007). Accumulating experimental evidence indicates that imperatorin inactivates gamma-aminobutyric acid (GABA)-transaminase and thus increases the GABA level in the brain (Choi et al. 2005). In the context of the present experiment, it is important to note that this compound inhibits acetylcholinesterase (AChE) activity (Loizzo et al. 2008). It is established that the cholinergic system is involved in memory function (Blake et al. 2014). Cholinergic deficiency in Alzheimer’s disease (AD), particularly in the basal forebrain, seems to be an important factor in producing dementia (Buckingham et al. 2009; Sugaya et al. 1990). Experimental evidence shows that the activity of AChE is significantly increased in the cerebral cortex, hippocampus, and amygdala in patients with AD (Serrano-Pozo et al. 2011). Various drugs from the group of acetylcholinesterase inhibitors (AChEI) (e.g., donepezil, galanthamine, metrifonate, and rivastigmine) can cause cognitive improvement, which depends on their level of inhibition of the cholinesterase activity (Loizzo et al. 2008). However, there are many limitations on AChEI use, e.g., adverse cholinergic side effects, hepatotoxicity, or poor bioavailability (Rountree et al. 2009). Moreover, it is important to note that oxidative stress is believed to be a critical factor in AD, and also inflammatory and immune mechanisms may play a role in the degenerative process in this disease (Ding et al. 2007). The central nervous system (CNS) is very susceptible to oxidative stress as the brain has a high consumption of oxygen, contains large amounts of free-radical generating iron and substances like ascorbate, glutamate, and polyunsaturated fatty acids, that easily undergo redox-reaction leading to radicals’ formation and exhibits relatively poor antioxidant defense systems (Walton et al. 2012). Additionally, coumarins, as well as their derivatives, were found to exert antioxidant and anti-inflammatory effects (Kostova 2006; Vianna et al. 2012).

Therefore, the main purpose of this study was to investigate for the first time the influence of imperatorin on scopolamine-induced memory impairment in the passive avoidance (PA) paradigm. It is established that scopolamine induces memory impairment associated with attenuation of cholinergic neurotransmission, as well as an increases of processes connected with oxidative stress in the brain (Fan et al. 2005). The activities of antioxidant enzymes superoxide dismutase (SOD), glutathione peroxidase (GPx), and glutathione reductase (GR), as well as the concentration of malondialdehyde (MDA), within the brain tissue, were determined to evaluate possible protective effect of imperatorin on scopolamine-induced oxidative stress.

## Materials and methods

### Animals

The experiments were carried out on naive male Swiss mice (Farm of Laboratory Animals, Warszawa, Poland) weighing 20–25 g at the beginning of the experiments. The animals were maintained under standard laboratory conditions (12 h light/dark cycle, room temperature 21 ± 1 °C) with free access to tap water and laboratory chow (Bacutil, Motycz, Poland), and were adapted to the laboratory conditions for at least 1 week. Each experimental group consisted of 8–12 animals. All experiments were carried out according to the National Institute of Health Guidelines for the Care and Use of Laboratory Animals and to the European Community Council Directive for the Care and Use of Laboratory Animals of 24 November 1986 (86/609/EEC), and were approved by the local ethics committee. The different mice were used for each drug and time treatment.

### Drugs

The following compounds were tested: scopolamine (Sigma-Aldrich, St. Louis, MO, USA), rivastigmine tartrate (Tocris Bioscience, UK), and imperatorin (8-isopentenyloxypsoralen [9-(3-methylbut-2-enyloxy)-7H-furo[3,2-g]chromen-7-one]. Imperatorin was extracted from fruits of *Angelica officinalis* (*Angelica archangelica*, Apiaceae) collected in August 2011 in Medicinal Plant Garden, Department of Pharmacognosy, Medical University of Lublin, Poland. The air-dried and powdered fruits (10 g) were extracted under reflux with methanol. The extract was separated with spectrum high-performance counter-current chromatograph (Dynamic Extractions, Slough, UK) equipped with semi-preparative coil with 137 ml capacity. System composed of heptane, ethyl acetate, methanol, and water (1:1:1:1, *v/v*) was used for separation, the upper phase was used as a stationary phase. The apparatus was rotated at 1,600 rpm, the mobile phase was pumped into the column at a flow rate of 6.0 ml/min, and the effluent from the coil was monitored at 254 nm. Eight hundred milligrams of the crude methanol extract was dissolved in 6 ml of two phase solvent system and injected into the column through the 6 ml injection valve. Fractions were collected in every minute. Imperatorin (0.095 g) with the purity of more than 99 % was detected in fractions 30–40. The identity and purity of imperatorin were confirmed by RP-HPLC and ^1^H NMR analyses.

Scopolamine was dissolved in a saline solution (0.9 % NaCl). Imperatorin was suspended in a 1 % solution of Tween 80 (Sigma, St. Louis, MO, USA) and also dissolved in a saline solution. Drugs were administered intraperitoneally (i.p.) at the volume of 10 ml/kg. Fresh drug solutions were prepared on each day of experimentation. Control groups received saline injections of the same volume and via the same route of administration.

### Passive avoidance task

According to Venault et al. (1986), the step-through passive avoidance task may be recognized as a measure of long-term memory. The apparatus consisted of a two-compartment acrylic box with the lighted compartment (10 × 13 × 15 cm) and the darkened compartment (25 × 20 × 15 cm). The light chamber was illuminated by a fluorescent light bulb (8 W) and was connected to the dark chamber which was equipped with the electric grid floor. Entrance of animals to the dark box was punished by an electric foot shock (0.2 mA for 2 s).

On the first day of training (pretest), mice were placed individually into the light compartment and allowed to explore the light box. After 30 s, the guillotine door was raised to allow the mice to enter the dark compartment. When the mice entered the dark compartment, the guillotine door was closed and an electric foot shock (0.2 mA) of 2 s duration was delivered immediately to the animal via the grid floor. The latency time for entering the dark compartment was recorded (TL1). If the mouse failed to enter the dark box within 300 s, it was placed into this dark box, the door was closed, and the electric foot shock was delivered to the animal. In this case, TL1 value was recorded as 300 s.

In the subsequent trial (retention), 24 h later, the same mice were again placed individually in the light compartment of the PA apparatus. After a 30-s adaptation period in the light (safe) chamber, the door between the compartments was raised and the time taken to reenter the dark compartment was recorded (TL2). No foot shock was applied in this trial. If the animal did not enter the dark compartment within 300 s, the test was stopped and TL2 was recorded as 300 s (Allami et al. 2011; Javadi-Paydar et al. 2012). The experimental procedure involved the examination of both memory acquisition (formation of memory traces, the animals received substances prior to the pretest) and memory consolidation (animals received injections of the substance after the pretest).

### Locomotor activity

Locomotor activity of mice was measured with photoresistor actimeters (circular cages, diameter 25 cm, two light beams). The animals were placed individually in the actimeter for 60 min. The number of crossings the light beams by the mice was recorded as the locomotor activity.

#### Treatment

The doses of imperatorin, rivastigmine, and scopolamine were chosen based on literature data (Deiana et al. 2009; Gupta et al. 2012; Łuszczki et al. 2007) and our recently published article (Budzynska et al. 2012, 2013; Kruk et al. 2011).

### Memory-related behavior

During the acute treatment, the animals were allocated to the following drug groups: saline, rivastigmine (0.5 mg/kg, i.p.), scopolamine (1 mg/kg, i.p.), imperatorin (1, 5, 10 mg/kg, i.p.), or imperatorin coadministered with scopolamine. To measure the memory acquisition processes, scopolamine was administered 20 min before the pretest, whereas imperatorin and rivastigmine were administered 30 min before the pretest. To measure the memory consolidation processes, rivastigmine or scopolamine (1 mg/kg) was administered immediately after the pretest, whereas imperatorin was administered 15 min after pretest or after scopolamine injection. On the second day, the mice were retested.

In the second set of the experiments, animals were randomly allocated to receive 6 days of i.p. injections of imperatorin (1, 5, and 10 mg/kg, i.p.) or saline, twice daily (8:00 a.m. and 8:00 p.m.). On the seventh day, these animals were subjected to saline, scopolamine (1 mg/kg, i.p.), imperatorin (1, 5, and 10 mg/kg, i.p.), or imperatorin coadministered with scopolamine. To measure the memory acquisition processes, scopolamine was administered 20 min before the pretest and imperatorin 30 min before the pretest. To measure the memory consolidation processes, scopolamine (1 mg/kg) was administered immediately after the pretest, whereas imperatorin was administered 15 min after the pretest or after scopolamine injection. On the eighth day, the mice were retested.

### Locomotor activity

The animals were allocated to the following drug groups: saline, scopolamine (1 mg/kg, i.p.), and imperatorin (1, 5, and 10 mg/kg, i.p.) administered alone or 10 min before saline or scopolamine (1 mg/kg) injection. Immediately after the last injection, animals were placed into the actimeters.

### Tissues collection and preparation

Following the PA test conducted after repeatable administration of imperatorin mice was anesthetized, the decapitated and the whole brain were carefully withdrawn and rinsed in icy-cold isotonic saline to remove the blood. The cerebral cortex and hippocampus were rapidly dissected. Whole brains (*n* = 10), as well as the cerebral cortex (*n* = 10) and hippocampus (*n* = 10), were used for the study.

The collected tissues were homogenized in 10 volumes of 20 mM Tris–HCl buffer (pH 7.4) on ice for 20 s and centrifuged at 12,000 g for 30 min at 4 °C. The supernatant was collected and used for further study. Activities of the antioxidant enzymes (SOD, GPx, and GR) and MDA level were determined from these supernatants spectrophotometrically with the use of HITACHI 2800 apparatus.

### Biochemical estimations

#### Determination of protein content

The protein content was determined by the Bradford method (Bradford 1976) using BSA as the standard.

### Determination of SOD activity

The activity of SOD was measured with the use of ready-to-use diagnostic kits RANSOD by Randox. The method employs xantine and xantine oxidase (XOD) to generate superoxide radicals, which react with iodonitrotetrazolium chloride to form red formazan dye. The superoxide dismutase activity is then measured by the degree of inhibition of the reaction. The increase in absorbance at 505 nm is read.

### Determination of GPx activity

The activity of GPx was measured with the use of ready-to-use diagnostic kits RANSEL by Randox. This method is based on that of Paglia and Valentine (Paglia and Valentine 1967). GPx catalyzed the oxidation of glutathione (GSH) by cumene hydroperoxide. In the presence of glutathione reductase (GR) and NADPH, the oxidized glutatione (GSSG) is immediately converted to the reduced form with a concomitant oxidation of NADPH to NADP^+^. The decrease in absorbance at 340 nm is measured.

### Determination of GR activity

The activity of GR was measured with the use of ready-to-use diagnostic kits GLUT RED by Randox. The principle of the method (Goldberg and Spooner 1983) is based on the reaction of oxidized glutathione (GSSG) reduction in the presence of NADPH, which is oxidized to NADP^+^, catalyzed by glutatione reductase. The decrease in absorbance is measured at 340 nm.

### Estimation of lipid peroxidation products

MDA was measured by the thiobarbituric acid (TBA) reaction (Ledwozyw et al. 1986). Briefly, 0.5 ml of tissue homogenate supernatant was mixed with 2.5 ml 1.22 M TCA in 0.6 M HCl and allowed to stand for 15 min. Then 1.5 ml of 0.9 % TBA was added, and the mixture was incubated for 30 min in a boiling water bath. After cooling, 4 ml of *n*-butanol was added and the mixture was shaken variously. The samples were centrifuged at 1,500 g for 10 min, and then the absorbance of organic phase was measured at 532 nm with respect to blank (*n*-butanol alone). The concentration of MDA was read from the standard curve obtained by using malonaldehyde bis-dimethylacetal.

### Statistical analysis

The data were expressed as the means ± standard error of the mean (S.E.M). The statistical analyses were performed by the two-way analysis of variance (ANOVA). Post hoc comparison of means was carried out with the Tukey’s test for multiple comparisons, when appropriate. The confidence limit of *p* < 0.05 was considered a statistically significant.

For the memory-related behaviors, the changes in PA performance were expressed as the difference between retention and training latencies, and were taken as an index of latency (IL). IL was calculated for each animal and reports as the ratio:

$$ \mathrm{IL} = \mathrm{TL}2\hbox{-} \mathrm{TL}1/\mathrm{TL}1 $$

TL1: the time taken to enter the dark compartment during the training
TL2: the time taken to reenter the dark compartment during the retention (Chimakurthy and Talasila 2010).

## Results

### Single imperatorin and rivastigmine injection effects on memory-related processes in PA test in mice

One-way ANOVA revealed that, at the acquisition trial, acute administration of imperatorin (1, 5, and 10 mg/kg) and rivastigmine (0.5 mg/kg) changed IL values [F(4,39) = 6.008; *p* = 0.0009]. Indeed, the post hoc Tukey’s test showed that imperatorin at the doses of 5 and 10 mg/kg (*p* < 0.05 and <0.01, respectively) and rivastigmine at the dose of 0.5 mg/kg (*p* < 0.001) significantly increased IL as compared with the saline-treated mice (Table 1). Similarly, at the consolidation trial, the acute doses of imperatorin (5 and 10 mg/kg) and rivastigmine (0.5 mg/kg) changed IL, as compared with the saline-treated mice ([F(4,38) = 6.024; *p* = 0.0009], one-way ANOVA). Indeed, the post hoc Tukey’s test revealed a statistically significant effects: *p* < 0.05 for imperatorin (5 and 10 mg/kg) and rivastigmine (0.5 mg/kg) (Table 1), indicating that both drugs, at the used doses, improved this stage of memory and learning processes.

**Table 1:** Effects of acute administration of imperatorin (IMP; 1, 5, and 10 mg/kg) and rivastigmine (RIVA; 0.5 mg/kg, i.p) on the memory acquisition or consolidation trials using the PA test in mice. Appropriate groups of mice received saline, imperatorin (1, 5, or 10 mg/kg, i.p.), or rivastigmine (RIVA; 0.5 mg/kg, i.p) before the pretest (acquisition) or after the pretest (consolidation). Data represent the means ± SEM and are expressed as latency index (IL); *n* = 8–12; *p < 0.05, **p < 0.01, ***p < 0.001 versus saline-treated group; Tukey’s test

	saline	RIVA	IMP	IMP	IMP	F	*p* value
		0.5 mg/kg	1 mg/kg	5 mg/kg	10 mg/kg		
ACQUISITION							
IL	2.180 ± 0.294	8.200 ± 0.204***	2.854 ± 0.396	4.847 ± 0.350*	4.999 ± 0.188**	F(4,39) = 6.008	0.0009
CONSOLIDATION							
IL	2.389 ± 0.129	7.201 ± 1.845***	1.530 ± 0.388	4.352 ± 0.384*	4.701 ± 0.338**	F(4,38) = 6,024	0.0009

### Single imperatorin injection effects on memory-related processes induced by scopolamine in PA test in mice

**Fig. 1:**
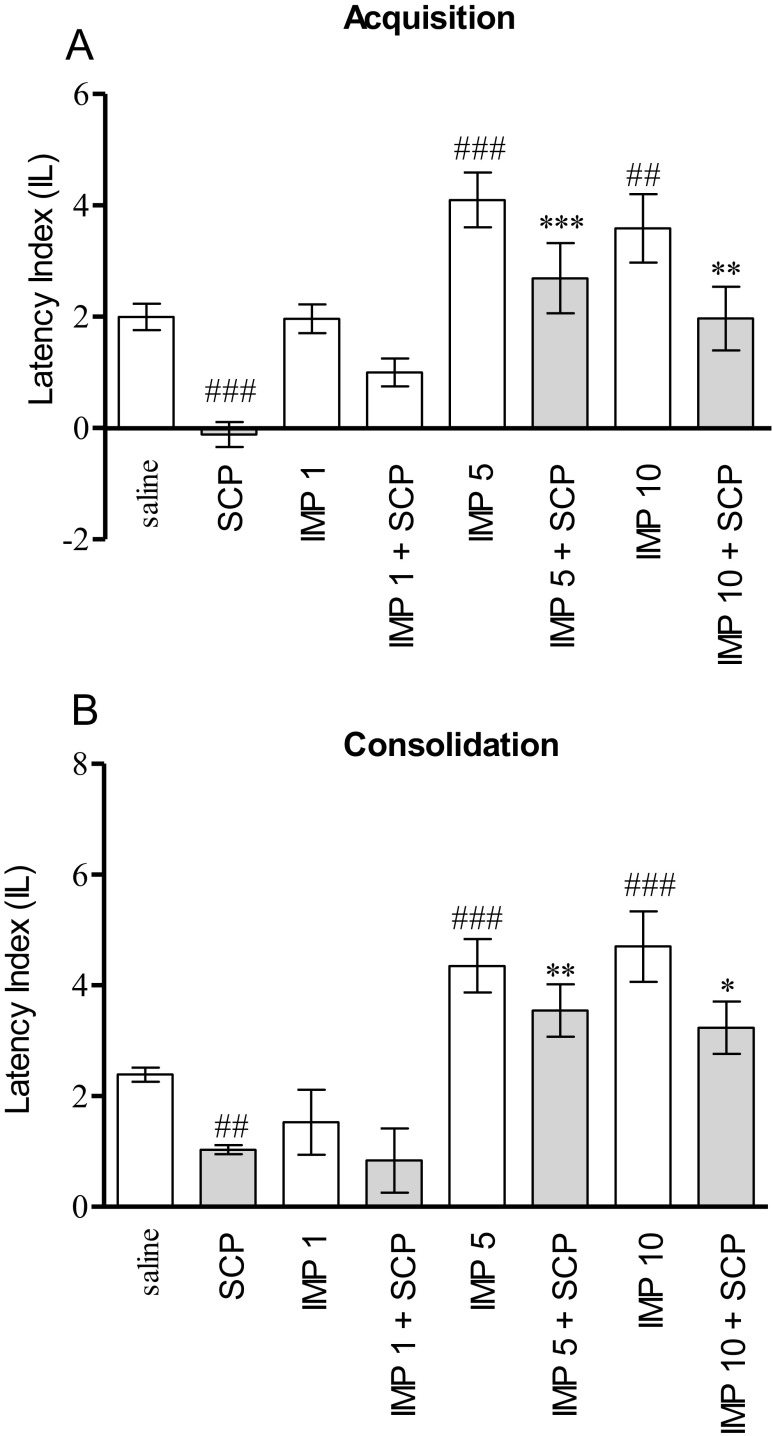
Effects of acute administration of imperatorin (IMP; 1, 5, or 10 mg/kg) on the memory acquisition trial (**a**) or consolidation trial (**b**) induced by scopolamine (SCP; 1 mg/kg), using the PA test in mice. Appropriate groups of mice received saline, imperatorin (1, 5, or 10 mg/kg, i.p.), or scopolamine (1 mg/kg, i.p.), coadministered with saline or scopolamine (1 mg/kg, i.p*.*) before the pretest (**a**) or immediately after the pretest (**b**). Data represent the means ± SEM and are expressed as the latency index (IL); *n* = 8–12; the means ± SEM; ^##^ *p* < 0.01, ^###^ *p* < 0.001 versus saline-treated control group; *p < 0.05, **p < 0.01; ***p < 0.001 versus scopolamine-treated control group; Tukey’s test

Figure 1a indicates the effects of injection of scopolamine (1 mg/kg) and imperatorin (1, 5, and 10 mg/kg, i.p.) alone or in combination on memory acquisition during the retention trial in PA task (two-way ANOVA; pretreatment [F(3,45) = 30.61, *p* < 0.0001], treatment [F(1,45) = 21.43, *p* < 0.0001] without interactions effect [F(3,45) = 2.65, *p* = 0.060]). In scopolamine-treated group, there was a significant decrease in the IL value as compared with the saline-treated mice (*p* < 0.001), indicating that scopolamine at the used dose impaired acquisition of memory and learning. Furthermore, the post hoc Tukey’s test revealed a statistically significant improvement in cognitive processes in the animals administered with imperatorin (5 and 10 mg/kg) (*p* < 0.001 and <0.01, respectively) as compared with the saline-treated mice. Additionally, administration of imperatorin at the doses of 5 and 10 mg/kg prevented the scopolamine-induced decrease of IL values as compared with the scopolamine group (*p* < 0.001 and <0.01, respectively).

For memory consolidation during the retention trial, two-way ANOVA revealed a statistically significant effect caused by the administration of scopolamine and imperatorin (pretreatment [F(3,42) = 16.89, *p* < 0.0001], treatment [F(1,42) = 9.19, *p* < 0.0042] without interactions effect [F(3,42) = 0.29, *p* = 0.8306]). The post hoc Tukey’s test showed that scopolamine significantly impaired memory consolidation (*p* < 0.01), whereas imperatorin given alone at the doses of 5 and 10 mg/kg improved cognitive processes (*p* < 0.001) as compared with the saline-treated mice. Moreover, the post hoc Tukey’s test revealed a statistically significant effect in memory and learning processes in the animals administered with scopolamine (1 mg/kg) and imperatorin (5 and 10 mg/kg, i.p.) or in combination (p < 0.01 and <0.05, respectively) (Fig. 1b).

### Repeated imperatorin injection effects on memory-related processes induced by scopolamine in PA test in mice

**Fig. 2:**
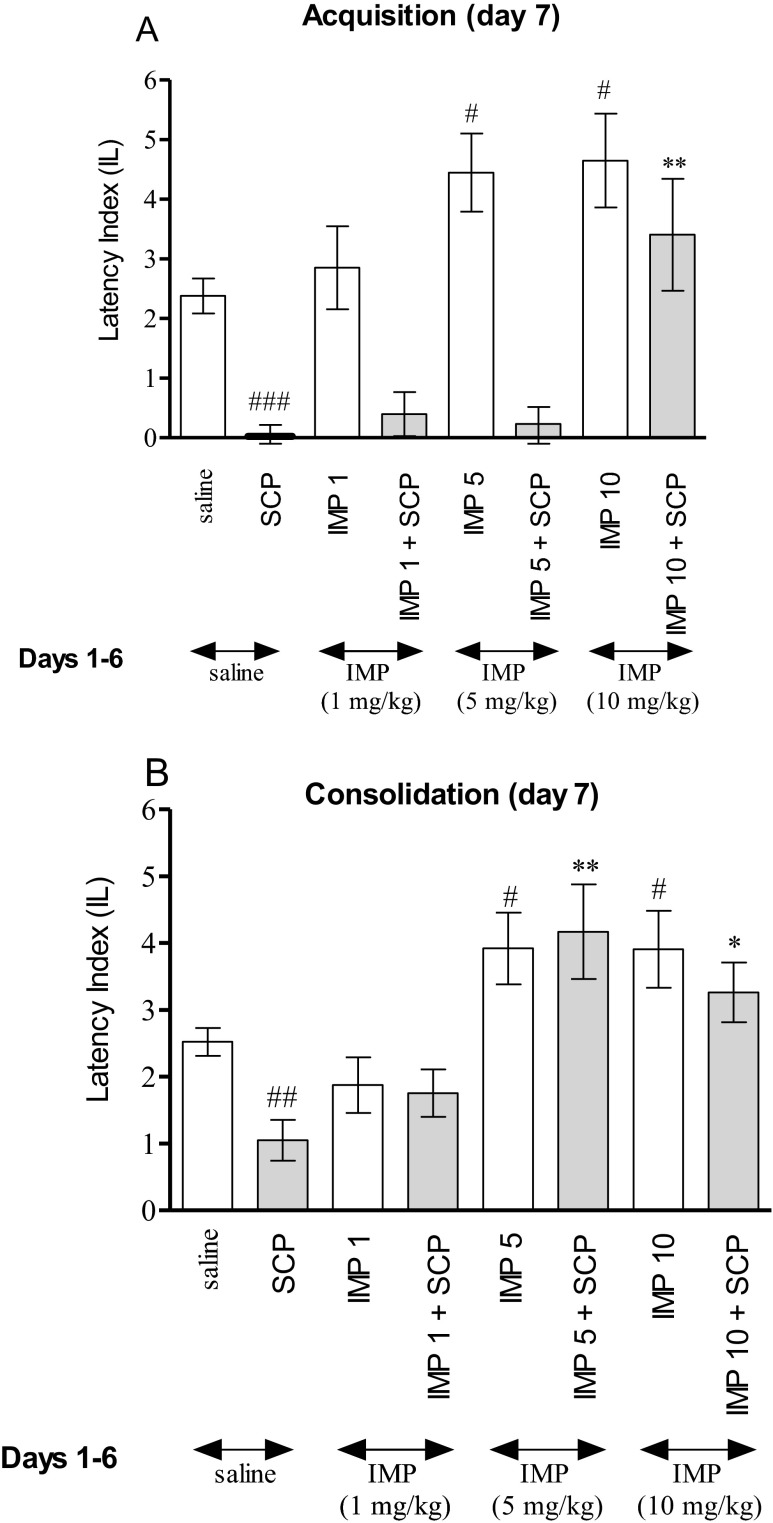
Effect of subchronic administration of imperatorin (IMP; 1, 5, or 10 mg/kg) on the memory acquisition trial (**a**) or consolidation trial (**b**) induced by scopolamine (SCP; 1 mg/kg), using the PA test in mice. Imperatorin (1, 5, or 10 mg/kg) or saline was administered for 6 days, twice daily. On the seventh day, appropriate group of mice received saline, imperatorin (1, 5, or 10 mg/kg, i.p.), or scopolamine (1 mg/kg, i.p.), coadministered with saline or scopolamine (1 mg/kg, i.p*.*) before the pretest (**a**) or immediately after the pretest (**b**). Data represent the means ± SEM and are expressed as latency index (IL); *n* = 8–12; the means ± SEM; ^*#*^ *p* < 0.05, ^##^ *p* < 0.01, ^###^ *p* < 0.001 versus saline-treated control group; *p < 0.05, **p < 0.01 versus scopolamine-treated control group; Tukey’s test

Figure 2a indicates the effects of repeated injections of imperatorin on memory acquisition impaired by scopolamine during the retention trial in PA task (two-way ANOVA; pretreatment [F(3,41) = 7.10, *p* = 0.0006], treatment [F(1,41) = 45.95, *p* < 0.0001] without interactions effect [F(3,41) = 2.62, *p* = 0.0635]). The post hoc Tukey’s test revealed that imperatorin given repeatedly at the doses of 5 and 10 mg/kg significantly increased IL value as compared with the saline-treated mice, thus indicating that subchronic administration of imperatorin improved acquisition of the memory and learning processes during the retention trial (*p* < 0.05). In contrast, in mice treated subchronically with saline and on seventh day with scopolamine (1 mg/kg), we observed impairment of memory acquisition (*p* < 0.001) as compared with the saline-treated mice. Furthermore, the post hoc Tukey’s test revealed a statistically significant improvement in memory and learning processes in the animals administered repeatedly with imperatorin (10 mg/kg) and on the seventh day injected with imperatorin in combination with scopolamine (*p* < 0.01) versus the scopolamine-treated mice.

As it is shown in Fig. 2b, at the consolidation trial, two-way ANOVA indicated that the mice treated for 6 days, twice daily with imperatorin (1, 5, and 10 mg/kg) and on the seventh day administered with imperatorin in combination with scopolamine (1 mg/kg), demonstrated change of IL values (treatment [F(1,36) = 12.55, *p* < 0.0001] without pretreatment effect [F(3,36) = 2.23, *p* = 0.1442] and interactions effect [F(3,36) = 1.26, *p* = 0.3040]).

The post hoc Tukey’s test showed that injection of imperatorin on seventh day at the doses of 5 and 10 mg/kg improved cognitive processes (*p* < 0.05) in rodents, as compared with the saline-treated mice. Additionally, subchronically treatment with saline and on seventh day with scopolamine decreased memory consolidation (*p* < 0.01). It was also revealed that repeated administration of imperatorin at the doses of 5 and 10 mg/kg significantly improved impairment of memory and learning processes induced by acute injection of scopolamine (*p* < 0.01 and <0.05, respectively), as compared with the subchronic saline and on the seventh day scopolamine-treated mice (Fig. 2b).

### Locomotor activity

The effect of imperatorin and combined administration of imperatorin and scopolamine on locomotor activity in mice is shown in Table 2. Two-way ANOVA indicated changes in locomotor activity after 30 min (treatment [F(1,52) = 63.34, *p* < 0.0001] without pretreatment effect [F(3,52) = 0.92, *p* = 0.4380] and interactions effect [F(3,52) = 0.92, *p* = 0.4380] and after 60 min (treatment [F(1,52) = 82.28, *p* < 0.0001] without pretreatment effect [F(3,52) = 0.25, *p* = 0.8584] and interactions effect [F(3,52) = 0.50, *p* = 0.6831]. The post hoc Tukey’s test showed that scopolamine significantly increased locomotor activity measured 30 and 60 min after administration as compared with the saline-treated mice (p < 0.001 and <0.01, respectively). Moreover, imperatorin given acutely at the used doses and after appropriate periods of time did not affect locomotor activity in mice. Combined administration of imperatorin and scopolamine did not significantly increase the locomotor activity counted after 30 and 60 min, when compared with scopolamine-treated group.

**Table 2:** Effect of scopolamine (SCP; 1 mg/kg, i.p) and imperatorin (IMP; 1, 5, and 10 mg/kg, i.p) administered separately or in combination on spontaneous locomotor activity in mice. Mice were injected with imperatorin 10 min before scopolamine administration and then immediately placed in actimeters. Locomotor activity (number of interruptions of light beams) was recorded for the 30 and 60 min. Data are presented as the means ± SEM. *n* = 8–12, **p < 0.01, ***p < 0.001 versus saline-treated group; Tukey’s test

	Saline	SCP	IMP 1 mg/kg	IMP 5 mg/kg	IMP 10 mg/kg	IMP 1 mg/kg + SCP	IMP5 mg/kg + SCP	IMP10 mg/kg + SCP
Photocell beam breaks ± SEM (30 min)	375.80 ± 25.16	863.30 ± 188.70***	450.30 ± 41.55	524.00 ± 19.60	426.10 ± 39.12	744.60 ± 89.56	886.10 ± 64.71	923.50 ± 34.50
Photocell beam breaks ± SEM (60 min)	717.80 ± 66.08	1,421.00 ± 306.40**	644.10 ± 47.31	846.50 ± 54.66	709.30 ± 89.75	1,557.00 ± 106.10	1,490.00 ± 103.60	1,497.00 ± 73.68

### Oxidative stress indicators

Table 3 presents the effect of subchronic imperatorin (1, 5, or 10 mg/kg, i.p.) administration and single scopolamine (1 mg/kg, i.p.) injection, separately or in combination, on SOD activity within (i) the whole brain (two-way ANOVA; pretreatment [F(1,88) = 11.21, *p* = 0.0012] without treatment effect [F = (3,88) = 0.38, *p* = 0.7681] and interactions effect [F(3,88) = 0.25, *p* = 0.8629]), (ii) the cortex (two-way ANOVA; pretreatment [F(1,88) = 16.92, *p* < 0.0001] and interactions effect [F(3,88) = 5.39, *p* = 0.0018] without treatment effect [F = (3,88) = 2.13, *p* = 0.1018]), and (iii) the hippocampus ([two-way ANOVA; pretreatment [F(1,88) = 10.87, *p* < 0.0014], interactions [F(3,88) = 4.42, *p* = 0.0061] without treatment effect [F = (3,88) = 2.49, *p* = 0.0654]) of mice. No significant changes in SOD activity were found in the whole brain of experimental animals, while the post hoc Tukey’s test showed that scopolamine administration significantly decreased SOD activity in examined brain structures: the cortex (*p* < 0.001) and hippocampus (*p* < 0.001). In the cortex, imperatorin administered with scopolamine was found to increase the activity of SOD at the doses of 5 and 10 mg/kg (*p* < 0.05), and in the hippocampus, this furanocoumarin injected at the dose of 10 mg/kg increased the level of SOD reduced by administration of scopolamine.

**Table 3:** Effect of subchronic administration of imperatorin (IMP; 1, 5, or 10 mg/kg, i.p.) and single scopolamine injection (SCP; 1 mg/kg i.p.) on the seventh day, separately or jointly, on activity of SOD in the whole brain, cortex and hippocampus of mice. Data are presented as the means ± SEM; *n* = 8–12; ***p < 0.001 versus saline-treated control group, ^#^ *p* < 0.05 versus scopolamine-treated control group; Tukey’s test

Treatment	SOD [U/mg protein]		
	Brain	Cortex	Hippocampus
Saline	2.78 ± 0.09	13.86 ± 0.89	12.97 ± 0.73
SCP	2.38 ± 0.14	9.68 ± 0.75***	9.38 ± 0.46***
IMP 1 mg/kg	2.96 ± 0.15	13.65 ± 0.29	11.06 ± 0.53
IMP 1 mg/kg + SCP	2.43 ± 0.16	12.38 ± 0.65^#^	9.76 ± 0.59
IMP 5 mg/kg	2.79 ± 0.16	13.41 ± 0.60	11.49 ± 0.61
IMP 5 mg/kg + SCP	2.49 ± 0.19	12.88 ± 0.46^#^	11.00 ± 0.22
IMP 10 mg/kg	2.89 ± 0.17	13.87 ± 0.61	11.97 ± 0.83
IMP 10 mg/kg + SCP	2.62 ± 0.17	13.29 ± 0.33^#^	12.10 ± 0.88^#^

Changes in GPx activity in (i) the whole brain ([two-way ANOVA; pretreatment [F(1,88) = 29.00, *p* < 0.001] without treatment effect [F(3,88) = 2.57, *p* = 0.0592] and interactions [F(3,88) = 1.80, *p* = 0.1525]), (ii) the cortex ([two-way ANOVA; pretreatment [F(1,88) = 14.52, *p* < 0.0003], treatment effect [F(3,88) = 4.26, *p* = 0.0074], and interactions [F(3,88) = 3.44, p = 0.0203]), and (iii) the hippocampus ([two-way ANOVA; pretreatment [F(1,88) = 98.05, *p* < 0.0001] and interactions [F(3,88) = 7.03, *p* = 0.0003], treatment effect [F(3,88) = 4.20, *p* = 0.0080]) are presented in Table 4. In scopolamine-treated group, significant decrease in GPx activity was noticed in the whole brain, as well as both examined brain structures like the prefrontal cortex and hippocampus (*p* < 0.001), in comparison with saline-treated control group. Moreover, the post hoc Tukey’s test indicated increasing in GPx activity in the cortex when imperatorin at the doses of 5 and 10 mg/kg was administered before scopolamine injection (p < 0.01 and <0.05, respectively), as compared with scopolamine-treated control group. Also, the changes were statistically significant in case of the highest dose of imperatorin (10 mg/kg) in the hippocampus (*p* < 0.01).

**Table 4:** Effect of repeated administration of imperatorin (IMP; 1, 5, or 10 mg/kg, i.p.) and single scopolamine injection (SCP; 1 mg/kg, i.p.) on the seventh day, separately or jointly, on activity of GPx in the whole brain, cortex and hippocampus of mice. Data are presented as the means ± SEM; *n* = 8–12; ***p < 0.001 versus saline-treated control group, ^#^ *p* < 0.05, ^##^ *p* < 0.01 versus scopolamine-treated control group; Tukey’s test

Treatment	GPx [U/g protein]		
	Brain	Cortex	Hippocampus
Saline	15.87 ± 0.55	64.29 ± 1.88	62.48 ± 2.88
SCP	10.07 ± 1.01***	48.01 ± 2.49***	32.91 ± 2.27***
IMP 1 mg/kg	12.99 ± 0.45	65.66 ± 2.65	62.14 ± 2.19
IMP 1 mg/kg + SCP	11.26 ± 0.99	53.93 ± 2.62	32.40 ± 3.21
IMP 5 mg/kg	14.91 ± 0.93	67.29 ± 4.42	55.03 ± 4.00
IMP 5 mg/kg + SCP	12.38 ± 0.87	67.94 ± 1.50^##^	41.56 ± 2.24
IMP 10 mg/kg	15.77 ± 0.96	63.36 ± 4.70	61.68 ± 2.99
IMP 10 mg/kg + SCP	13.42 ± 0.49	63.36 ± 4.17^#^	52.00 ± 3.47^##^

Two-way ANOVA analysis revealed a statistically significant effect of scopolamine administration in GR activity within the prefrontal cortex (pretreatment [F(1,88) = 38.13, *p* < 0.0001], treatment [F(3,88) = 3.86, *p* = 0.0121] without interactions effect [F(3,88) = 2.09, *p* < 0.1078]) and the hippocampus (pretreatment [F(1,88) = 47.86, *p* < 0.0001] without treatment effect [F(3,88) = 0.21, *p* = 0.8926] and interaction effect [F(3,88) = 0.73, *p* = 0.5373]) as presented in Table 5. The post hoc Tukey’s test showed that scopolamine injection caused a significant decrease in GR activity in the cortex (*p* < 0.001) and hippocampus (*p* < 0.001) as compared with saline-treated control group. Additionally, the highest dose of imperatorin (10 mg/kg) administered subchronically before scopolamine (1 mg/kg) injection was found to cause a significant increase in GR activity in the prefrontal cortex (*p* < 0.05) as compared with scopolamine-treated control group.

**Table 5:** Effect of repeated administration of imperatorin (IMP; 1, 5, or 10 mg/kg, i.p.) and single scopolamine injection (SCP; 1 mg/kg, i.p.) on the seventh day, separately or jointly, on activity of GR in the cortex and hippocampus of mice. Data are presented as the means ± SEM; *n* = 8–12, ***p < 0.001 versus saline-treated control group; ^#^ *p* < 0.05 versus scopolamine-treated control group; Tukey’s test

Treatment	GR [U/g protein]	
	Cortex	Hippocampus
Saline	53.29 ± 4.78	98.73 ± 3.15
SCP	34.63 ± 12.95***	78.63 ± 2.86***
IMP 1 mg/kg	46.87 ± 1.22	90.01 ± 4.63
IMP 1 mg/kg + SCP	35.38 ± 1.48	77.80 ± 2.05
IMP 5 mg/kg	51.46 ± 2.46	95.13 ± 3.91
IMP 5 mg/kg + SCP	41.48 ± 1.62	76.89 ± 4.59
IMP 10 mg/kg	52.61 ± 2.39	93.40 ± 2.83
IMP 10 mg/kg + SCP	46.93 ± 1.91^#^	82.16 ± 4.06

The effect of scopolamine and imperatorin, as well as their combined administration in the concentration of MDA, the main product of lipids peroxidation, is presented in Table 6. Two-way ANOVA indicated changes in MDA level in the cortex (pretreatment [F(1,88) = 251.16, *p* < 0.0001] and interactions [F(3,88) = 21.91, *p* < 0.0001] without treatment effect [F(3,88) = 1.40, *p* = 0.2474]) and the hippocampus (pretreatment [F(1,88) = 54.77, *p* < 0.0001] without treatment effect [F(3,88) = 0.14, *p* = 0.9360] and interaction effect [F(3,88) = 2.36, *p* = 0.0772]) of the experimental animals. The post hoc Tukey’s test showed that scopolamine significantly increased MDA concentration in the cortex and hippocampus as compared with saline-treated mice (*p* < 0.001). Moreover, all doses of imperatorin (1, 5, and 10 mg/kg) administered repeatedly before scopolamine injection caused decrease in MDA level in the cortex (*p* < 0.001) versus scopolamine-treated group. The values of MDA concentration in the cortex after combined administration of scopolamine and imperatorin were statistically increased (1 and 5 mg/kg; *p* < 0.001, 10 mg/kg; *p* < 0.05) as compared with imperatorin-treated groups. Also, the post hoc Tukey’s showed that imperatorin significantly increased MDA concentration in the cortex as compared with saline-treated mice (5 mg/kg; *p* < 0.05, 10 mg/kg; *p* < 0.01). Additionally, subchronic administration of imperatorin at the doses of 5 and 10 mg/kg before scopolamine (1 mg/kg) injection was found to cause a significant decrease in MDA activity in the hippocampus (*p* < 0.05) as compared with scopolamine-treated control group.

**Table 6:** Effect of subchronic administration of imperatorin (IMP; 1, 5, or 10 mg/kg, i.p.) and single scopolamine injection (SCP; 1 mg/kg, i.p.) on the seventh day, separately or in combination, on concentration of MDA in the cortex and hippocampus of mice. Data are presented as the means ± SEM; *n* = 8–12; *p < 0.05, **p < 0.01, ***p < 0.001 versus saline-treated control group, ^#^ *p* < 0.05, ^###^ *p* < 0.001 versus scopolamine-treated control group, &*p* < 0.05, &&&*p* < 0.001 versus imperatorin-treated control group; Tukey’s test

Treatment	MDA [μM/g wet w.]	
	Cortex	Hippocampus
Saline	10.64 ± 0.13	3.454 ± 0.30
SCP	23.52 ± 0.69***	6.187 ± 0.44***
IMP 1 mg/kg	12.34 ± 0.41	3.817 ± 0.25
IMP 1 mg/kg + SCP	19.59 ± 0.86^###,^ &&&	5.582 ± 0.28
IMP 5 mg/kg	13.76 ± 0.51*	3.986 ± 0.33
IMP 5 mg/kg + SCP	18.63 ± 0.87^###,^ &&&	5.699 ± 0.42^#^
IMP 10 mg/kg	14.30 ± 0.64**	4.196 ± 0.35
IMP 10 mg/kg + SCP	17.64 ± 0.57^###,^ &	5.121 ± 0.29^#^

## Discussion

In the present studies, imperatorin was examined for the first time in order to prove whether this compound could ameliorate memory dysfunction induced by scopolamine. Our results indicate that acute imperatorin injections at the doses of 5 and 10 mg/kg improved the memory acquisition and consolidation impaired by scopolamine (1 mg/kg). Moreover, only repeatable administration of the highest dose of imperatorin (10 mg/kg) significantly attenuated the effects of scopolamine on memory acquisition, whereas the doses of 5 and 10 mg/kg of furanocoumarin were effective when memory consolidation was measured. Furthermore, our results demonstrate the enhancement of locomotor activity after scopolamine injections. Scopolamine has been generally found to increase locomotor activity, already at the systemic doses of 0.056 mg/kg and higher (Chintoh et al. 2003; Gholamreza et al. 2002; Nomura et al. 1994). Thus, imperatorin at any used doses did not modify the locomotor activity of mice or scopolamine-induced effects. Our previous study has already shown that acute and repeated administration of imperatorin (10 and 20 mg/kg) improved different stages of memory processes (both acquisition and consolidation) in the modified EPM test, a different animal task used to measure cognitive effects in rodents (Budzynska et al. 2012). Our experiments also demonstrated that both acute and repeatable injections of the subthreshold dose of imperatorin (1 mg/kg) improved memory and learning processes when coadministered with the inactive dose of nicotine in the PA paradigm (Budzynska et al. 2013).

Imperatorin was shown to inhibit the activity of AChE, which is an enzyme responsible for degradation of acetylcholine (ACh), the neurotransmitter essential for cognitive function (Loizzo et al. 2008). Also, it is well established that the cholinergic neurotransmission system in the basal forebrain plays an important role in learning and memory, and thus maintaining ACh level is critical for the brain function (Blake et al. 2014). Moreover, memory deficits associated with AD may be due to the damage of the cholinergic pathways in the brain (Buckingham et al. 2009; Snowden et al. 2011). Thus, current therapeutic approach for treating AD is the AChE inhibitors that increase the availability of ACh in the central cholinergic synapses, such as donepezil, rivastigmine, and galantamine (Terry and Buccafusco 2003), as well as acting on the glutamatergic system by blocking NMDA-type glutamate receptors (memantine) (Rountree et al. 2009). However, new drugs are needed to treat patients with AD, because current drugs may exert side effects such as gastrointestinal disturbance or bradycardia (Rountree et al. 2009). Thus, in the context of the present research, it is also important to mention that adverse effects, like neurotoxicity, after the administration of imperatorin have been reported after higher doses (329 and 443 mg/kg) than used in the present experiments (Łuszczki et al. 2009). Moreover, chronic toxicological studies revealed that imperatorin administered orally (p.o.) once daily for four consecutive days at doses up to 150 mg/kg did not affect blood clotting and have little or no systemic (renal, hepatic, or pulmonary) toxicity in mice (Kleiner et al. 2002).

Scopolamine is an anticholinergic drug that antagonizes the muscarinic cholinergic receptors (mAChRs) (subtypes; M1 and M2). This agent is capable of producing deficits in the processes of learning acquisition and consolidation (Schmeller et al. 1995). Experimental data show that ACh levels are significantly reduced in scopolamine-treated mice (Giridharan et al. 2011). Scopolamine-induced amnesia is connected with increased oxidative stress in the whole brain, as well as in particular structures associated with memory and learning (Pachauri et al. 2012). This compound was also found to disturb metabolism especially for low molecular weight antioxidants like glutathione, and therefore intensify the level of lipids peroxidation within the brain (El-Sherbiny et al. 2003), which is particularly vulnerable to reactive oxygen species (ROS) action due to high abundance of highly oxidable polyunsaturated fatty acids, such as arachidonic acid and docosahexaenoic acid (Smith et al. 2007). Thus, scopolamine-induced amnesia is a very well established animal model of memory dysfunction, widely used to test potential drugs of anti-Alzheimer's properties (Kanwal et al. 2010).

Therefore, in the present studies, we used this animal model of AD, in which we provoked scopolamine-induced memory deficits, to investigate the pro-cognitive properties of imperatorin in the PA test. This test is a fear-motivated paradigm, useful for evaluating the effects of novel chemical molecules on learning and memory as well as studying the mechanisms involved in cognition, in small laboratory animal (Tsuji et al. 2003). In this model, the cognitive impairment was induced by scopolamine and imperatorin pretreatment was able to prevent the cognitive decline as the scopolamine-induced shortening of the step-through latency was successfully restored by imperatorin administered acutely and repeatable.

In the context of the present study, we can expect that the mechanism responsible for the cognitive improvement induced by imperatorin may depend on the inhibition of AChE activity. Many reports indicate that inhibited AChE activity leads to an increased ACh levels in the brain, especially in the central cortex and hippocampus, the two major areas involved in the cognitive processes (Fayuk and Yakel 2004). Thus, high ACh concentrations improved cognition in humans and in several memory and learning models in rodents (Canal and Imbimbo 1996; Knoppman 2001). Our study also showed that acute injection of AChEI, rivastigmine, improves acquisition and consolidation of cognitive processes in PA test, thereby affirming the important role of ACH in cognitive functions. As compared with imperatorin, this effect was more marked what may suggest the different degrees of inhibition of the enzyme by this compounds. However, our data may further suggest that the ameliorating effects of imperatorin on memory deficit might have resulted from the modulation of ACh levels through an inhibition of AChE enzyme.

Additionally, imperatorin administered subchronically has a different impact on scopolamine-induced memory impairment depending on phases of memory processes. This coumarin administered at the dose of 5 mg/kg attenuated scopolamine-induced memory consolidation impairment whereas did not improve impaired memory acquisition. These positive effects of imperatorin on the scopolamine-induced memory impairment during consolidation trial can be connected with the involvement of the different type of cholinergic receptors in the partial stages of memory. In behavioral studies on animals, it has been found that agonists of cholinergic muscarinic receptor, injected after earlier training, improve cognitive processes. Thus, it seems that stimulation of cholinergic receptors, especially muscarinic, is an important process for memory consolidation step not followed by acquisition phase (Power et al. 2003). Moreover, the data indicated that modulation of memory consolidation processes through administration of drugs after having a workout depends on small amygdala. The injection of muscarinic receptor agonist to amygdala after preceded by training improved the memory processes (McGaugh et al. 1993).

Another beneficial property of imperatorin found in the present study is the potential antioxidant activity. It is generally accepted that oxidative stress is significant in the formation of the pathology of AD. Experimental data suggest that during latent period of the disease the accumulation of oxidative damage processes can appear. This action leads to sudden appearance of clinical symptoms of AD, including cognitive deficits (Bonda et al. 2010). Several reports highlight the potential role of anti-oxidant therapeutic agents in the treatment of AD (Gilgun-Sherki et al. 2003).

It is well established that reactive oxygen species (ROS) are generated during normal metabolism. However, oxidative stress, which is an imbalance between ROS production and antioxidant capacity of an organism, is associated with numerous diseases (as cancer, cardiovascular disease, atherosclerosis, hypertension, etc.) also including neurodegenerative diseases (AD and Parkinson's disease) (Valko et al. 2007). Increased generation of free radicals can result from side effects of applied medical treatment, course of some diseases, and normal ageing of an organism.

Our paper confirms that scopolamine significantly increased MDA concentration, but we also revealed a strong impact of scopolamine administration on antioxidant enzymes activity, which was not stated previously (El-Sherbiny et al. 2003). The authors postulated scopolamine influence on glutathione level; however, they did not notice changes in GPx and SOD activity. Although we did not examined GSH level, our findings confirm scopolamine influences on that antioxidant through enzymes responsible for GSH turnover, i.e., GPx and GR, whose activities were significantly increased after scopolamine injection. Our study also revealed that scopolamine application did not cause significant changes in SOD activity in the whole brain, what was in accordance with the findings of El-Sherbiny et al. (2003). However, when particular brain structures connected with memory acquisition and consolidation were examined, a significant increase in SOD activity was stated in a group of mice which received a single amnesic dose of scopolamine in comparison with the saline-treated control group.

Coumarins, as well as their derivatives, were found to exert strong antioxidant and anti-inflammatory effects (Kostova 2006; Vianna et al. 2012). These compounds were stated to affect formation and scavenging of ROS and influence processes involving free radical-mediated injury in a similar way to flavonoids (Fylaktakidou et al. 2004). Many coumarins are recognized as lipoxygenase and cyclooxygenase inhibitors (Kontogiorgis et al. 2006), which are able to arrest or slow down pathological processes caused by increased activities of these enzymes, including production of neuroinflammation involving vasodilation and vasoconstriction, platelet aggregation, leukocyte chemotaxis and release of cytokines, and oxidative stress. Antioxidant properties of imperatorin were postulated by Cao et al. (2013) in the 2-kidney, 1-clip (2 K, 1C) model of hypertension used to investigate the potential antihypertensive effect. Other reports revealed that imperatorin inhibited superoxide dismutase (Raja et al. 2011), as well as decrease myeloperoxidase activity (Sun et al. 2012). Moreover, our previous studies also confirmed antioxidative properties of this coumarin. Imperatorin restrained nicotine-induced oxidative stress in examined brain structures, i.e., the hippocampus and cortex (Budzynska et al. 2013).

Our study revealed that treatment with imperatorin was found to reverse scopolamine-induced memory impairment in mice probably by reducing oxidative stress within the CNS. Our outcomes implied that subchronic administration of imperatorin was able to overwhelm pro-oxidant effects of scopolamine in the brain, i.e., an increase in antioxidant enzymes (SOD, GPx, and GR) activities and a decrease in MDA concentration. Interestingly, the changes were well-marked in single brain structures, the prefrontal cortex and hippocampus, rather than in the whole brain indicating specific scopolamine action in brain regions implicated in learning and memory processes.

However, according to our results, the antioxidant effect of imperatorin is dose-dependent and strongly correlated with memory and learning processes. The lowest dose (1 mg/kg) was found inactive, i.e., did not affected antioxidant barrier parameters as well as did not improved memory processes, while the higher doses (5 and 10 mg/kg) induced a statistically significant increase antioxidant enzymes activity and decrease MDA level as well as caused memory improvement in behavioral tests. The dose of imperatorin, 1 mg/kg, was also ineffective in the PA test. Coumarin administered acutely and subchronically at this dose did not influence cognitive processes, as well as did not improve memory and learning processes impaired by administration of scopolamine.

The current results do not provide a clear mechanism by which imperatorin affects memory processes. However, obtain results may suggest that imperatorin may be able to ameliorate memory impairments caused by cholinergic dysfunction through inhibition of AChE activity as well as inhibition of oxidative stress-related processes. The present studies demonstrated that acute and subchronic administration of imperatorin improved the scopolamine-induced memory acquisition and consolidation impairment in PA test in mice. Furthermore, we may conclude that observed antiamnestic effects of imperatorin may be attributed to antioxidant, and therefore neuroprotective properties of that furocoumarin. Currently, there is no cure for AD and additional preclinical studies may contribute to the development of more effective and devoided of significant side effects AD pharmacotherapy. Based on the present results, we can speculate that imperatorin may become a new treatment strategy which will change the course of the disease and improve the quality of life of people with dementia. Additionally, antioxidant prophylactics may be beneficial for people with a genetic risk factor for AD occurrence.
